# Exposure to Bisphenol A Correlates with Early-Onset Prostate Cancer and Promotes Centrosome Amplification and Anchorage-Independent Growth *In Vitro*

**DOI:** 10.1371/journal.pone.0090332

**Published:** 2014-03-03

**Authors:** Pheruza Tarapore, Jun Ying, Bin Ouyang, Barbara Burke, Bruce Bracken, Shuk-Mei Ho

**Affiliations:** 1 Department of Environmental Health, University of Cincinnati College of Medicine, Cincinnati, Ohio, United States of America; 2 Center for Environmental Genetics, University of Cincinnati College of Medicine, Cincinnati, Ohio, United States of America; 3 Cincinnati Cancer Center, University of Cincinnati College of Medicine, Cincinnati, Ohio, United States of America; 4 Department of Surgery, University of Cincinnati College of Medicine, Cincinnati, Ohio, United States of America; 5 Cincinnati Veteran Affairs Hospital Medical Center, Cincinnati, Ohio, United States of America; University of Kentucky College of Medicine, United States of America

## Abstract

Human exposure to bisphenol A (BPA) is ubiquitous. Animal studies found that BPA contributes to development of prostate cancer, but human data are scarce. Our study examined the association between urinary BPA levels and Prostate cancer and assessed the effects of BPA on induction of centrosome abnormalities as an underlying mechanism promoting prostate carcinogenesis. The study, involving 60 urology patients, found higher levels of urinary BPA (creatinine-adjusted) in Prostate cancer patients (5.74 µg/g [95% CI; 2.63, 12.51]) than in non-Prostate cancer patients (1.43 µg/g [95% CI; 0.70, 2.88]) (*p* = 0.012). The difference was even more significant in patients <65 years old. A trend toward a negative association between urinary BPA and serum PSA was observed in Prostate cancer patients but not in non-Prostate cancer patients. *In vitro* studies examined centrosomal abnormalities, microtubule nucleation, and anchorage-independent growth in four Prostate cancer cell lines (LNCaP, C4-2, 22Rv1, PC-3) and two immortalized normal prostate epithelial cell lines (NPrEC and RWPE-1). Exposure to low doses (0.01–100 nM) of BPA increased the percentage of cells with centrosome amplification two- to eight-fold. Dose responses either peaked or reached the plateaus with 0.1 nM BPA exposure. This low dose also promoted microtubule nucleation and regrowth at centrosomes in RWPE-1 and enhanced anchorage-independent growth in C4-2. These findings suggest that urinary BPA level is an independent prognostic marker in Prostate cancer and that BPA exposure may lower serum PSA levels in Prostate cancer patients. Moreover, disruption of the centrosome duplication cycle by low-dose BPA may contribute to neoplastic transformation of the prostate.

## Introduction

Prostate cancer (PCa) is the second most common malignancy among men in North America. Aging is a well-established risk factor for PCa [1]. One in six men will develop PCa over their lifetime; however, the cancer is rarely diagnosed in men <40 years old, with almost two-thirds cases reported [2], [3] in men at age 65. From 2006 to 2010, the median age at diagnosis was 66 years according to the statistics from National Cancer Institute's Surveillance Epidemiology and End Results Studies (2013) [4]. Major contributing factors other than age are race and family history [1], whereas little is known about the impact of endocrine disruptors on PCa.

Bisphenol A (BPA) is an organic compound with the chemical formula (CH_3_)_2_C(C_6_H_4_OH)_2_. BPA is used to make polycarbonate plastic and epoxy resins, which are present in thousands of consumer products [5], [6]. In the United States, exposure to BPA is widespread, exceeding 90% in the general population [7]. Dermal absorption, inhalation, and ingestion from contaminated food and water are the major routes of exposure [8]. As an endocrine disruptor that mimics estrogen and thyroid hormone, BPA also acts as a metabolic and immune disruptor. Thus, the adverse health effects of BPA are extensive [9], [10], and higher levels of BPA exposure correlate with increased risk of cardiovascular disease, obesity, diabetes, immune disorders, and a host of reproductive dysfunctions [11], [12], [13]. Moreover, *in vitro* and animal studies have shown that BPA exposure can increase the risk of mammary gland, brain, and prostate cancers [9]. However, human studies linking BPA exposure to heightened cancer risk are scarce. One such study in China showed that the incidence of meningioma was 1.6 times higher in adults with higher concentrations of BPA in urine than in those with lower concentrations [14]. Similar studies for PCa have not been available until now.

A centrosome comprises a pair of cylindrical structures called *centrioles* surrounded by pericentriolar material. Centrosomes are involved in organizing the interphase microtubule cytoskeleton, mitotic spindles, and cilia. Centrosome dysfunction (number and integrity), a hallmark of many cancers, is believed to initiate neoplastic transformation and promote disease progression [15], [16]. An abnormal number of centrosomes can result in mono- or multipolar mitosis, leading to increased aneuploidy [15], [16]. Another feature of centrosomal disruption is abnormalities in microtubule (MT) nucleation and anchoring. Such abnormalities were more frequently observed in breast cancer cells than in normal breast epithelial cells [15], [16]. Also, a significant number of genes associated with increased PCa risk are in pathways leading to centrosome dysfunction [17], [18]. These observations have prompted us to examine, in cell-based models, the adverse effects of BPA on the centrosome cycle as a mechanism contributing to prostate carcinogenesis.

We used a cross-sectional clinical study to examine the association between BPA exposure and PCa. We hypothesized that BPA plays a role in prostate carcinogenesis. We found that patients with PCa are more likely than those without PCa to have higher levels of BPA in their urine. We observed a trend toward a negative correlation between urinary BPA and serum PSA levels in PCa patients. We performed *in vitro* studies to assess the effects of BPA on centrosome number, the formation of MT asters, and colonization in soft agar in two immortalized normal prostate epithelial cell lines (RWPE-1 and NPrEC) and four PCa cell lines (LNCaP, C4-2, 22Rv1, PC-3). We found that the percentage of cells with centrosome amplification (CA) increased in response to low-dose BPA exposure and that the relationship was non-monotonic for most cell lines. Moreover, exposure to low-dose BPA promoted MT aster organization in the non-cancerous RWPE-1 and increased anchorage-independent growth in the androgen-independent C4-2 PCa cell line. In aggregate, these findings reveal a previously unknown relationship between BPA exposure and PCa and suggest a mechanism underlying the role of BPA in neoplastic transformation and disease progression.

## Materials and Methods

### Patients and the collection of urine samples

Patients were recruited from the urologic clinic at the University of Cincinnati Medical Center under a protocol approved by the University of Cincinnati Institutional Review Board. Table 1 lists patient characteristics and diagnostic information. After signing an informed consent form, patients underwent a digital rectal examination and were asked to provide a 20- to 50-ml urine specimen before their scheduled ultrasound-guided prostate biopsy. All procedures in this study were approved by the University of Cincinnati Institutional Review Board. Urine samples were centrifuged, the sediments were collected for a PCa biomarker study [19], and the supernatants were stored in aliquots at −80°C for BPA analysis. Among the 60 samples used for this study, 27 were from patients with PCa (PCa) and 33 were from patients without PCa (non-PCa).

**Table 1:** Summary of baseline characteristics (n = 60).

Variable	Category or unit	PCa (n = 27) Descriptive statistics*	Non-PCa (n = 33) Descriptive statistics*	*p* value†
Age	Year	69.67±10.29	62.76±7.15	0.003
Ln(serum PSA)	ng/mL	1.73±0.87	1.42±0.65	0.121
Gleason score	6 = 3+3	15 (71.4%)		
	7 = 3+4	4 (19.0%)		
	7 = 4+3	2 (9.5%)		
Treatment	Watchful waiting	14 (51.9%)		
	Prostatectomy	13 (48.1%)		
Rising PSA	No	23 (85.2%)		
	Yes	4 (14.8%)#		
Other cancer	No	25 (92.6%)		
	Yes	2 (7.4%)		
Recurrence	No	13 (85%)		
	Yes	2 (15%)		

*Numerical variables are summarized using mean ± standard deviation (SD). Categorical variables are summarized using frequency (in %).

† *p* values are calculated from t-tests.

#Serum PSA significantly rose during follow-up.

### Measurement of BPA in urine samples

BPA levels in samples were determined in the Laboratory of Organic Analytical Chemistry of Wadsworth Center, New York State Department of Health, (Albany, NY). High-performance liquid chromatography (HPLC) coupled with electrospray triple-quadrupole mass spectrometry (ESI-MS/MS) was used to quantify BPA, a technique similar to that described earlier, with some modifications [20], [21]. In brief, 500 µl of each urine sample was mixed with 1 ml of glucuronidase (2 µl/ml) for digestion and extraction. For quality control, 5 ng of ^13^C_12_-BPA was added to each mixture. Extracts were applied to an Agilent 1100 series HPLC interfaced with an Applied Biosystems API 2000 electrospray MS/MS (Applied Biosystems, Foster City, CA) for quantitative of BPA. Data were acquired using multiple-reaction monitoring for the transitions of 227>212 for BPA, and 239>224 for ^13^C_12_-BPA. The minimum detection limit (MDL) of BPA in this protocol was 0.05 ng/ml. For concentrations below the MDL, a value equal to the MDL divided by the square root of 2 was used in statistical analyses [22]. Reported concentrations were corrected for the recoveries of surrogate standard (isotopic dilution method). The BPA standard spiked to selected sample matrices and passed through the entire analytical procedure yielded a recovery of 88%±8% (mean ± SD). An external calibration curve was prepared by injecting 10 µl of 0.05, 0.1, 0.2, 0.5, 1, 2, 5, 10, 50, and 100 ng/ml standards, and the regression coefficient was 0.99.

### Normalization of urine BPA

Urinary creatinine levels were used to adjust for variability in dilution and to determine the validity of a spot urine sample for assessing chemical exposure [23]. A creatinine (urinary) assay kit from Cayman Chemical Company (Ann Arbor, MI) was used according to the manufacturer's protocol to measure urinary creatinine levels. The creatinine levels were used to adjust the urinary concentrations of BPA measured by the HPLC-ESI-MS/MS to obtain the “creatinine-adjusted” BPA levels (BPA levels) in µg/g.

### Cells

The PCa cell lines PC-3, LNCaP, C4-2, and 22Rv1 were obtained from the American Type Culture Collection (ATCC, Manassas, VA) and cultured under standard, recommended, conditions. A description of the origin of the immortalized normal prostate epithelial NPrEC cell line has been published [24]; the other immortalized normal prostate epithelial cell line, RWPE-1, was purchased from ATCC (Manassas, VA) and was grown in Defined Keratinocyte-SFM medium (Invitrogen, Carlsbad, CA) with growth-promoting supplement. Cell cultures were maintained at 37°C in a humidified incubator with a 5% CO_2_ atmosphere.

### BPA treatments

Cells from each cell line were seeded into six-well plates with glass cover slips at 25,000 cells/well. After 24 h, the medium was changed to phenol red–free media with 10% charcoal-stripped serum for another 24 h, at which time BPA was added to achieve a final concentration of 0, 0.01 nM, 0.1 nM, 1 nM, 10 nM, or 100 nM. The experiment was repeated five times to generate a total of five samples per cell line per BPA concentration.

### Indirect immunofluorescence

For immunostaining of centrosomes, cells were fixed with methanol for 5 min at −20°C and then processed for γ-tubulin (clone GTU88 antibody, Sigma Immunochemicals), α-tubulin (clone DM1A, Sigma Immunochemicals), and centrin (sc-50452, Santacruz Biotechnology) staining as previously described [25]. In brief, cells were extracted in 1% NP-40 in PBS for 10 min. Cells were probed with primary antibodies, and the antibody-antigen complexes were detected with Alexa fluor 488- or 594-conjugated antibodies (Molecular Probes). Cells were also stained for DNA with 4′, 6-diamidino-2-phenylindole (DAPI, Invitrogen). Immunostained cells were examined by fluorescence microscopy.

### Microtubule (MT) aster formation assay

The effect of BPA on microtubule dynamics was determined by an assay of MT aster formation described previously [25]. In brief, cells were treated with nocodazole (1.5 µg/ml) for 40 min on ice to depolymerize interphase MTs, washed with PBS to remove the nocodazole, and incubated in fresh warm medium for 10 min at 37°C to allow for MT regrowth.

### Measurements

The number of centrosomes per cell was scored by fluorescence microscopy. At least 150 cells were examined per treatment, and the percentage of cells with an abnormal number of centrosomes calculated from the total number of cells examined was used as the outcome measure for the analysis. A major abnormality in CA was defined as a cell with more than two centrosomes.

### Anchorage-independent growth assay

Cells were assayed for anchorage-independent growth by measuring the efficiency of colony formation in semisolid medium as described [26]. In brief, cells were cultured under conditions described above, in the presence or absence of 0.1 nM BPA, for ∼10 passages. We chose 0.1 nM because this concentration induced the highest percentage of cells with CA for most cell lines (see Results). Approximately 2,500 cells/35-mm well were embedded in soft agar. Cells were fed twice a week with fresh medium with and without BPA. After 2–3 weeks, colonies were counted under a microscope. Experiments were performed in triplicate and repeated twice. Colony-forming efficiency is the number of colonies obtained divided by the total number of cells plated, multiplied by 100.

### Statistical analysis

The primary measure in the clinical analysis was a continuous variable of urinary BPA level after normalization or adjustment for urinary creatinine level. Initial inspection of the distribution showed that this variable was highly skewed to the right. Hence, its log-transformed variable (LnBPA) was used as the dependent variable in the statistical models. The principal statistical model was a fixed-effect model to assess the association between the LnBPA and PCa status (1 = yes; 0 = no). We applied both unadjusted and adjusted methods to our fixed-effect model. In the unadjusted method, the PCa status was the only independent variable. In the adjusted method, we included age (stratified as age ≥65 vs. <65 years) and serum PSA levels as controlling covariates. We performed *post hoc* comparisons of means between PCa and non-PCa patients and a similar comparisons in subsets of patients stratified by age. Wilcoxon rank sum tests were used to validate the findings from the fixed-effect models to ensure that all their findings were robust (data not shown). For urinary BPA and other numeric independent variables such as serum PSA levels, the relationships were assessed with linear regression models and/or correlation coefficients.

In the *in vitro* analyses for each cell line, we used the fixed effect model to assess the association of the percentage of cells with CA to the BPA concentration used to treat the cells and *post hoc* analyses adjusted for multiple comparisons using a Bonferroni's test. The anchorage-independent growth assay data were analyzed by two-sample *t*-tests. All statistical tests were performed with an SAS 9.3 software (SAS, Cary, NC) package. *P*-values <0.05 were considered statistically significant.

## Results

### Urinary BPA level is associated with PCa and may have prognostic value

We studied 60 urology patients, 27 with PCa and 33 without PCa. The mean age (± standard deviation [SD]) of PCa patients was 69.7±10.3 yr (min, 56 yr; max. 87 yr); they were older than non-PCa patients, who were 62.8±7.15 yr (min. 46 yr; max. 77 yr; *p* = 0.003). Serum PSA levels of PCa and non-PCa patients were not different. The Gleason score of 71% of the PCa patients was 6, and 7 in the others. Their baseline characteristics are summarized in Table 1.

**Figure 1:**
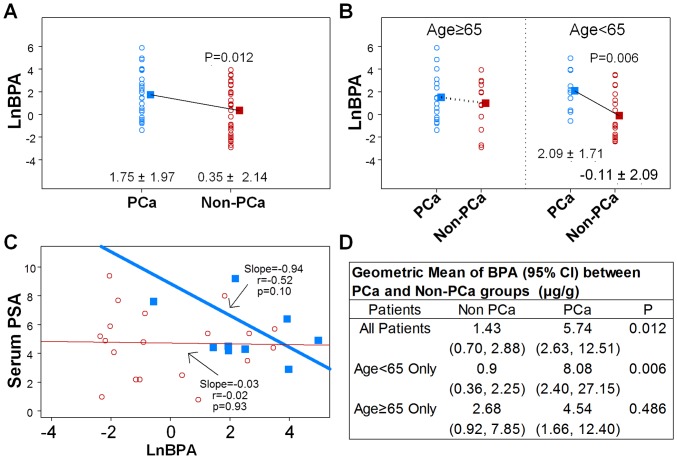
Scatter plots of LnBPA. Urine BPA levels are associated with PCa. The log-transformed BPA is referred to as LnBPA. Values in graph are mean ± SD of LnBPA. (A) Urine BPA levels are higher in PCa patients than in non-PCa patients. Means of LnBPA  = 1.75±1.97 in PCa (blue, n = 27) vs. 0.35±2.14 in non-PCa (red, n = 33), *p* = 0.012. (B) LnBPA in PCa vs. LnBPA in non-PCa, stratified by age = 65. Urine BPA levels are significantly higher in young PCa patients than in the respective non-PCa patients only in the age group <65 years old; *p* = 0.006. (C) Linear regression analyses of Serum PSA vs. LnBPA in patients <65 years old only (n = 30). Blue solid squares represent PCa patients; red inverse-circles represent non-PCa patients. Blue and red solid lines represent their regression lines, respectively. (D) Comparison of the geometric mean of BPA in PCa and non-PCa groups. The geometric mean (Geo) is defined as the exponential of the mean of LnBPA. Values are geometric means (95% CI) of BPA in unit of µg/g creatinine.

In all subjects (PCa and non-PCa), levels of urinary BPA were not associated with age and serum PSA and did not correlate with Gleason score of the cancer and cancer-related characteristics in PCa subjects (Table 2). However, patients with PCa had higher levels of urinary BPA (creatine adjusted), with a geometric mean of 5.74 [95% CI; 2.63, 12.51] µg/g (mean ± SD of LnBPA of 1.75±1.97), whereas the urinary BPA levels of non-PCa patients had a geometric mean of 1.43 [95% CI; 0.70, 2.88] µg/g (mean ± SD of LnBPA of 0.35±2.14; *p* = 0.012, Fig. 1A & 1D). Stratified analyses showed that the positive association was significant only among the 30 urologic patients younger than 65 (mean and median age  = 58 yr, minimum age  = 46 yr). In the younger patients (<65 yr), the geometric mean of urinary BPA levels among PCa patients was 8.08 [95% CI; 2.40, 27.15] µg/g (mean ± SD of LnBPA of 2.09±1.71) vs. a geometric mean of 0.90 [95% CI; 0.36, 2.25] µg/g (mean ± SD of LnBPA of −0.11±2.09) among non-PCa patients (*p* = 0.006; Fig. 1B & 1D). Moreover, linear regression analyses of this younger group revealed a trend toward a negative association between urinary BPA levels and serum PSA concentrations in the PCa patients (n = 10, r = −0.52, *p* = 0.10) but no such trend in non-PCa patients (Fig. 1C). The correlation did not reach significance at the 5% level because of the small sample size.

**Table 2:** Summary of LnBPA (log-transformed BPA) values and cancer-related characteristics (n = 27) for PCa patients.

Factor	Category	n	Mean ± SD	*p* value
Gleason score	6 = 3+3	15	1.49±0.44	0.595
	7 = 3+4	4	0.54±0.85	
	7 = 4+3	2	0.94±1.20	
Treatment	Watchful waiting	14	1.68±0.54	0.848
	Prostatectomy	13	1.83±0.56	
Rising PSA	No	23	1.89±0.41	0.367
	Yes	4	0.91±0.99	
Other cancer	No	25	1.66±0.40	0.435
	Yes	2	2.82±1.41	
Recurrence	No	25	1.81±0.40	0.558
	Yes	2	0.94±1.41	

### Low doses of BPA promoted centrosome amplification (CA)

**Figure 2:**
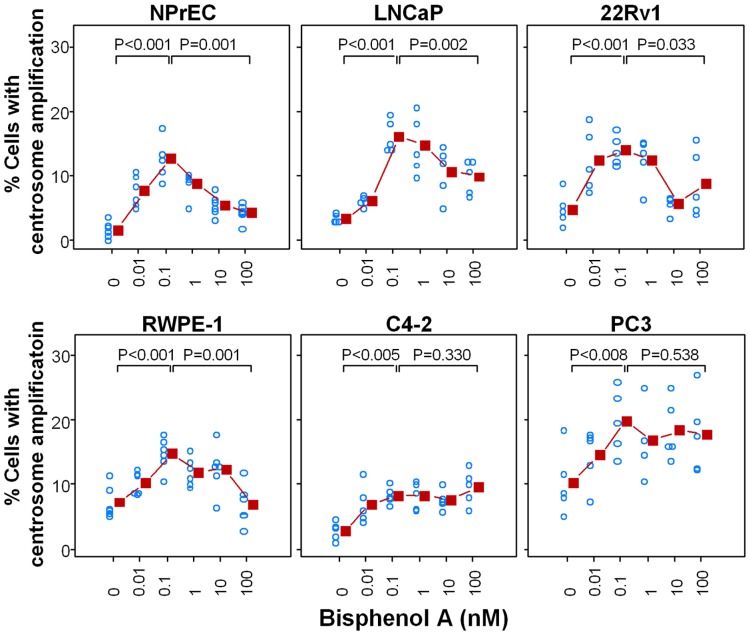
Low doses of BPA have an adverse effect on centrosome numbers in prostate cancer cells. The cell lines NPrEC, RWPE1, LNCaP, C4-2, 22Rv1, and PC3 were treated with medium containing 10% CSS plus 0, 0.01 nM, 0.1 nM, 1 nM, 10 nM and 100 nM BPA for 72 h. Cells were fixed with 100% cold methanol and immunostained for centrosomes and nuclei. The number of centrosomes per cell was scored by fluorescence microscopy. The results are shown as an average determined from five separate experiments. The scatter plot was generated of the percentage of cells with an abnormal number of centrosomes in response to BPA. Analyses was performed using a fixed effect model for each cell line. *Post hoc* comparisons of means were adjusted using Bonferroni's tests. The fold change is the percentage of cells with abnormal centrosomes at 0.1 nM BPA/the percentage of cells with abnormal centrosomes at 0 nM BPA.

**Figure 3:**
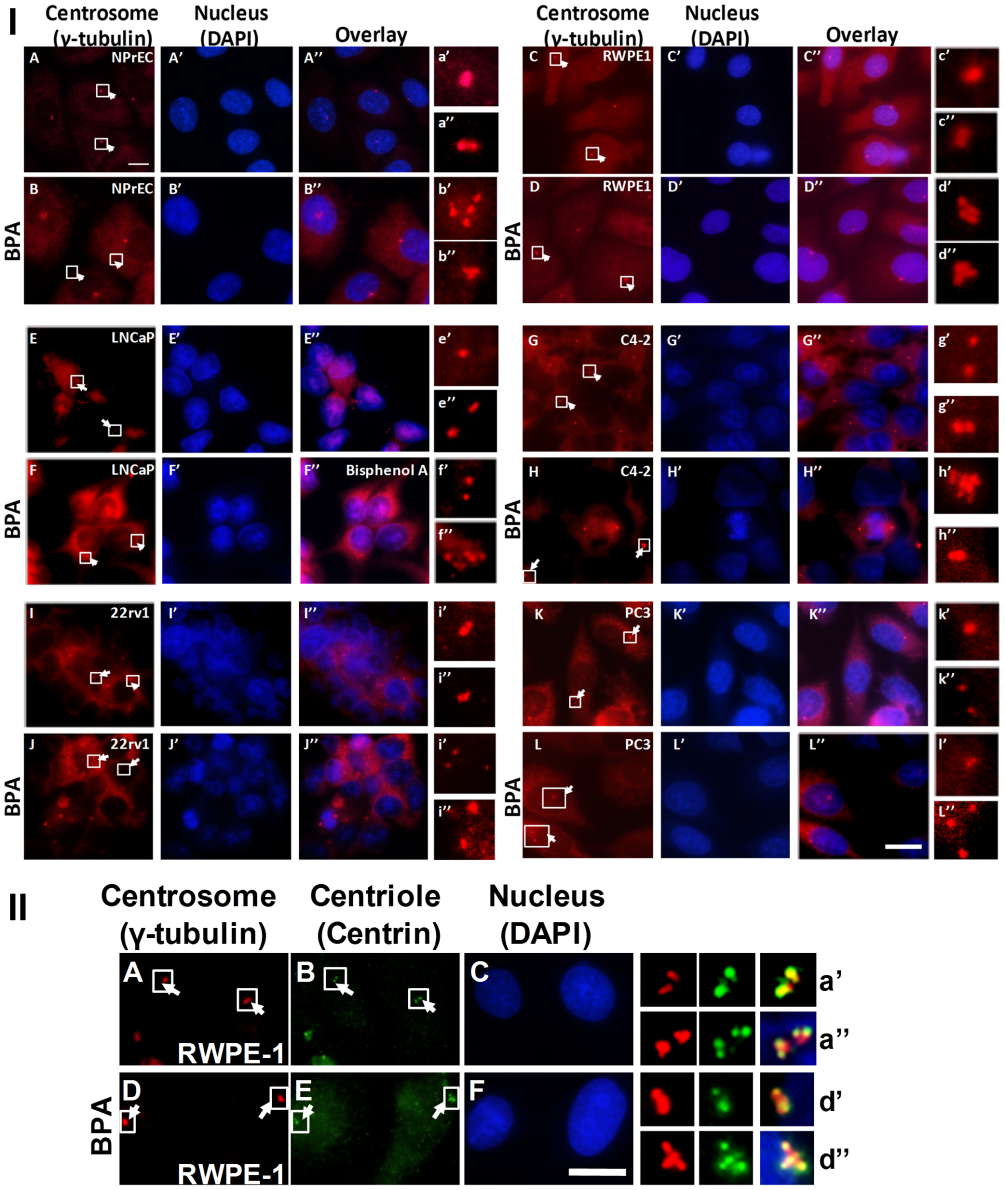
An increase in centrosome numbers is seen in prostate cancer cells exposed to BPA. (I) An increase in centrosome numbers. The cell lines NPrEC, RWPE1, LNCaP, C4-2, 22Rv1 and PC3 were treated with medium containing 10% CSS plus 0 or 0.1 nM BPA for 72 h. Cells were fixed with 100% cold methanol and immunostained for centrosomes (anti-γ-tubulin, red) and nucleus (DAPI, blue). The cells were examined by fluorescence microscopy. Arrows point to the positions of centrosomes, and panels on the right show magnified images of the indicated areas. Scale bar, 10 µm. (II) Centrosome amplification in the presence of BPA is not due to centriole separation. RWPE-1 cells were treated with 0.1 nM BPA for 3 days. Cells were fixed and immunostained for centrosomes (anti-γ-tubulin, red), centrioles (anti-centrin, green), and nucleus (DAPI, blue). Arrows point to the positions of centrosomes. Panels on right show magnified images of the indicated areas. Scale bar, 10 µm.

CA is commonly observed in human tumors and is a major factor contributing to chromosome instability [15], [27]. Depending on whether the cell is in the G1 or S/G2/M phase of the cell cycle, normal cells show one or two centrosomes, respectively. We determined whether treating cells with BPA changed the number of centrosomes, by treating cell cultures with increasing concentrations of BPA (0.01–100 nM) (Figs. 2 and 3). Untreated cells that served as controls showed the expected normal centrosome profile, in which most of the cells (>90%) contained either one or two centrosomes (Fig. 3-I, panels A, C, E, G, I, K). The untreated NPrEC had the fewest cells with centrosomal aberrations (1.7%), followed by C4-2 (2.9%), LNCaP (3.5%), 22Rv1 (4.9%), RWPE-1 (7.3%), and PC-3 (10.4%) (Fig.2). In contrast, all cell lines treated with BPA showed an increase (two- to eight-fold, Table 3) in the number of cells with three or more centrosomes (Fig. 2, Fig. 3-I panels B, D, F, H, J, L). The dose-response curves of the two non-cancerous cell lines, NPrEC and RWPE-1, and two PCa cell lines, LNCaP and 22Rv1, reveal a non-monotonic (biphasic) response relationship, with the maximal response with 0.1 nM BPA (Fig. 2). On the other hand, the two other PCa lines, C4-2 and PC-3, displayed an increasing dose-response curve that plateaus at the same low concentration of BPA (0.1 nM) (Fig. 2). The immortalized non-cancerous prostate epithelial cell line NPrEC-1, showed the highest fold change (mean ± SD, 8.1±2.4) in centrosome profile (Table 3), suggesting that its centrosome duplication cycle may be most sensitive to the effects of low-dose BPA on the promotion of CA.

**Table 3:** Fold change in the percentage of cells with centrosomal amplification in presence of 100

Cell line	Mean fold change ± SE (from BPA = 0 to BPA = 0.1 nM)*	*p* value of indicated cell lines vs. NPrEC-1†
NPrEC-1	8.1±2.4	-
LNCaP	4.7±0.5	0.047
C4-2	3.8±1.1	0.013
22RV1	3.6±0.8	0.009
RWPE-1	2.2±0.3	0.001
PC3	2.1±0.3	0.001

*Fold change is defined as % of cells with abnormal centrosomes at 0.1 nM BPA/% cells with abnormal centrosomes in untreated cells.

† *Post hoc* comparisons were performed under a fixed effect model and adjusted using Bonferroni's methods. Only the p-values of comparing NPrEC-1 to other cell lines are presented. Other comparisons between the cell lines were not statistically different.

### Low-dose BPA did not affect centriole splitting

Structurally, the centrosome consists of a pair of cylindrical structures called centrioles that act as the duplicating units. To verify the integrity of the centrosomes, we immunostained cells for centrin, a major constituent of the centriole cylinder, allowing visualization of the centriole pair within the centrosome. Fig. 3 shows representative images for RWPE-1 cells. Each dot detected by antibody to γ-tubulin (Fig. 3-II; panels A and D) was resolved to a pair of dots (representing a centriole pair) revealed by antibody to centrin at a higher magnification (Fig. 3-II; panels B and E, panels a, a″; d′, d″). These data thus indicate that the centrosomes are intact, containing a pair of centrioles. The centrosome profiles determined by counting the centrin signal were similar to those determined by counting the γ-tubulin signal (Fig. 2). Results for LNCaP, C4-2, 22Rv1, and NPrEC cells were similar. Hence, BPA had no effects on centrosome separation or centriole splitting.

### Low-dose BPA enhanced MT aster formation

**Figure 4:**
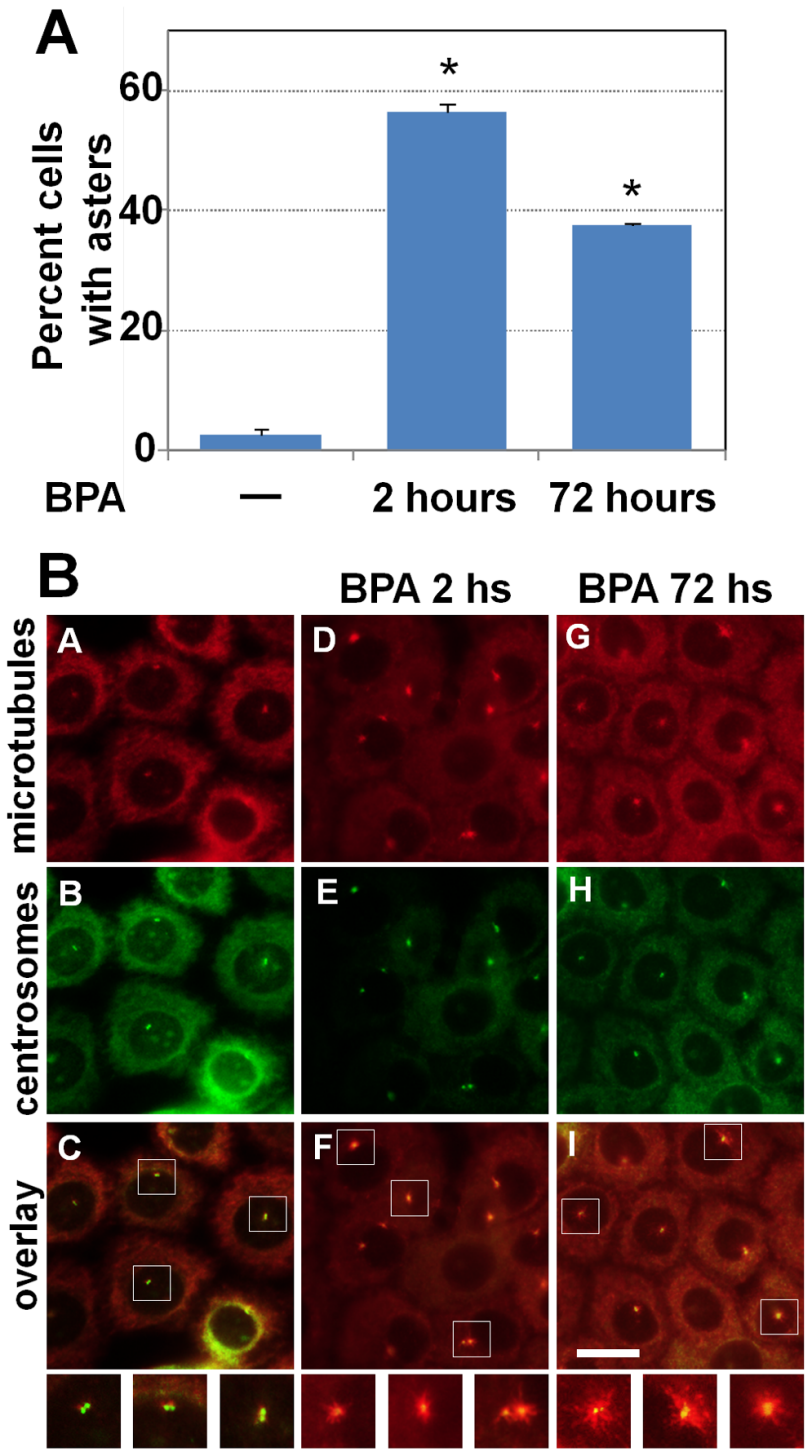
BPA enhances centrosomal aster formation. The microtubule aster formation assay was performed 2-immunostained for centrosomes (anti-γ-tubulin, red) and MTs (anti-α-tubulin, green). The centrosomal aster formation was assessed as positive if centrosomes had an MT aster with more than 15 MTs. The results shown in (A) are the average ± standard error (SE) from three experiments. For each experiment, >200 cells were examined. Significance was calculated using Student's t-test vs. 0 pM. *p≤0.00002.

The anchoring of MTs and their subsequent elongation to form radial MT arrays (asters) are critical events during interphase and also lead to the formation of the mitotic spindle associated with normal centrosome function [28]. RWPE-1 prostate cells assayed for MT aster formation (Fig. 4). Cells were first treated with nocodazole on ice to completely depolymerize interphase MTs; nocodazole was then removed, and cells were incubated in fresh warm medium for MT regrowth. The ability of the centrosomes to nucleate, anchor, and elongate MTs was determined by co-immunostaining for centrosomes (anti-γ-tubulin) and MTs (anti-α-tubulin). The MT aster forming activity of centrosomes was assessed according to the previously established protocol [25]. Untreated RWPE-1 cells showed negligible aster formation. After acute 2-h treatment with 0.1 nM BPA, short asters were seen 56% of cells. Three days post-treatment with BPA (chronic exposure), ∼37% cells showed asters (Fig. 4A, 4B panels g–i). Our data thus indicate that BPA enhances MT aster formation.

### Chronic BPA exposure promotes anchorage-independent growth in C4-2 cells

**Figure 5:**
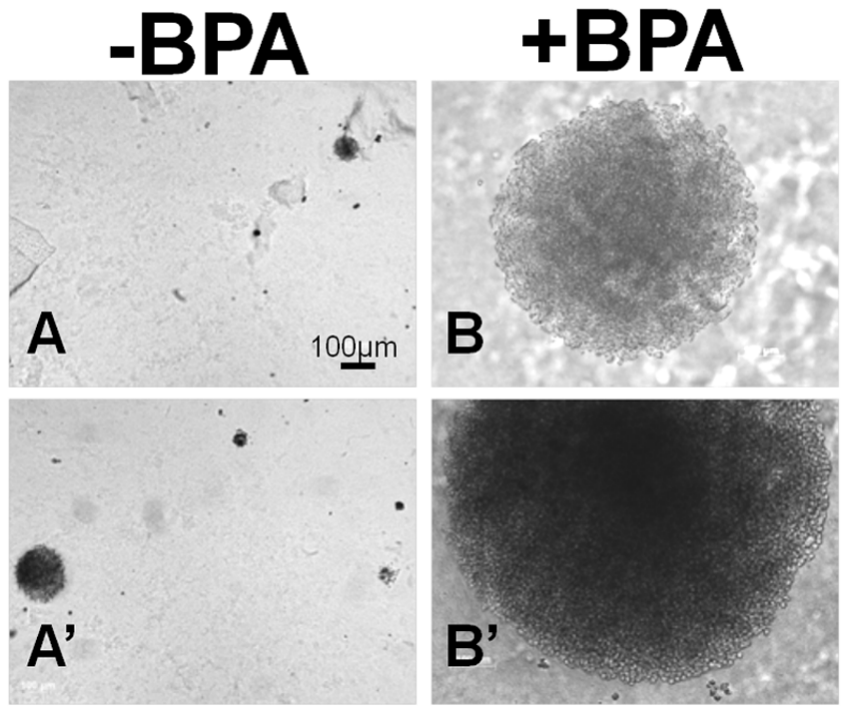
Cells grown in the absence and presence of 0.1-independent growth. Representative pictures of colonies after 2 weeks of incubation in agar. C4-2 cells in the presence of 0.1 nM BPA formed larger colonies (B, B′, 100–1200 µm diameter) compared with those grown in the absence of BPA (A, A′, 50–400 µm diameter).

The ability of chronic BPA exposure to transform or promote malignant growth of NPrEC, RWPE-1, LNCaP, and C4-2 cells was determined by a soft-agar colony-formation assay. The cells were grown in medium with or without 0.1 nM BPA for 10–14 passages before they were seeded on soft agar. Colony formation for NPrEC, RWPE-1, and LNCaP was <2%, and exposure to 0.1 nM BPA did not change the efficiency of colony formation. However, BPA-exposed C4-2 cells produced substantially more, larger, faster-growing soft-agar colonies (Table 4, Fig. 5). The percent efficiency of colony formation (mean ± SD) increased to 19.25±7.05% with BPA treatment compared with 2.03±0.40% in unexposed controls (*p*<0.001). The colony diameter was 50–400 µm in controls vs. 100–1,200 µm in BPA-treated C4-2 cells.

**Table 4:** Anchorage- independent growth in the presence and absence of BPA.

	Mean ± SD of anchorage- independent growth (% colonies)		
Cell type	Control (n = 6 replicates)	Exposed to BPA (n = 6 replicates)	*p* value*
RWPE1	0.10±0.05	0.13±0.07	0.397
NPrEC	0.15±0.08	0.21±0.18	0.504
LNCaP	0.64±0.29	0.60±0.25	0.836
C4-2	2.03±0.40	19.25±7.05	<0.001

*p values were computed using t-tests.

## Discussion

Evidence that BPA exposure contributes to PCa was derived from animal studies [29], [30], [31], [32] or cell-based [33], [34], [35], [36] models. To the best of our knowledge, this is the first study that provides preliminary evidence of an association of BPA exposure with PCa in a clinical setting. Our findings in 60 urologic patients show that urinary BPA level is an independent prognostic biomarker of PCa, as higher urinary BPA levels were detected in the 27 PCa patients (geometric mean, 5.74 [95% CI; 2.63, 12.51] µg/g creatinine) as compared with those in the 33 non-PCa patients (geometric mean, 1.43 [95% CI; 0.70, 2.88] µg/g creatinine) (*p* = 0.012). The detection limit for this study was 0.05 ng/ml. Several population studies have now established BPA as a ubiquitous environmental contaminant detectable in the urine of most individuals in US populations. In the first large-scale cross-sectional study in the US involving 2,517 participants of the 2003–2004 National Health and Nutrition Examination Survey (NHANES) [7], BPA was detected in 93% of the population at a geometric mean and a 95^th^ percentile concentration of 2.6 µg/g and 11.2 µg/g, respectively; the limit of detection was 0.4 ng/ml vs. 0.05 ng/ml in our study. A later study of 2,747 adult participants in the 2003–2006 NHANES [37] reported a geometric mean of 2.05 µg/g creatinine (25^th^ percentile: 1∶18, 75^th^ percentile: 3.33); the lower limit of detection was 0.36 ng/ml. Thus, the geometric mean of urinary BPA levels in the PCa patients in the present study was ∼2–2.5 times higher than the geometric means of those in large US cross-sectional studies. In contrast, the geometric mean in the non-PCa patients in this study was ∼50% lower than the geometric means of these two population studies.

A strength of this study is our use of the method recommended by the Centers for Disease Control and Prevention involving solid-phase extraction coupled with isotope dilution-HPLC-MS/MS to measure total urinary BPA in a reference laboratory. Furthermore, we corrected for variations caused by factors that affect urinary dilution by expressing our data relative to urinary creatinine concentrations. Finally, all patients had biopsy-confirmed, rather than self-reported, PCa. A potential limitation of our study was that total urinary BPA in our patients was measured only once. However, according to current literature, total urinary BPA concentrations (free plus conjugated) in spot samples (one-time measurement) is a reliable method of evaluating baseline exposure from all sources across time when the sample size is sufficiently large [38]. Although toxicokinetic studies have shown that BPA and its major metabolite, BPA-glucuronide, have rather short half-lives (∼2.5 h) in the bloodstream and that they are rapidly excreted with urine [39], [40], cross-sectional population studies have suggested substantially longer half-lives due to nonfood exposure, bioaccumulation in body tissues such as fat, and liver function, especially those related to glucuronidation of BPA [12], [39]. The presence of high BPA concentrations in urine may suggest that the lifestyle habits of these patients may sustain higher levels of exposure. In this regard, in one clinical study, BPA levels in urine samples collected on the same day from male and female partners correlated [41], supporting the premise that similar lifestyle choices may determine the level of BPA exposure. Moreover, a recent study showed higher within-person variability (over 1–3 years) in BPA levels as compared with the total variability in 80 women [42]. Collectively, these studies highlight the significance of our finding that a one-time sampling of urinary BPA correlates with PCa.

Stratified analyses showed that the association between urinary BPA levels and PCa is highly significant (*p* = 0.006) among the 30 patients <65 years old (mean and median age  = 58, minimum age  = 46) but that this association does not reach significance among the half of patients >65 years (Fig. 1). These findings are intriguing, but perplexing. Taken at face value, they suggest that higher BPA exposure is associated with earlier onset of PCa. However, on the basis of the theory of developmental reprogramming of cancer risk [43], our findings raise the possibility of early-life reprogramming of PCa in humans. In rat studies, neonates fed environmentally relevant levels of BPA had an increased risk of developing prostate neoplasms [29], [44]. According to this reasoning, one should note that the younger PCa patients were either just born or young children when BPA was introduced for commercial use in the US in 1957. For example, the patient aged 64 years old would have been around 11 years old (prepuberty) when first exposed to BPA and those younger might have been exposed *in utero*.

Further analyses of the age groups <65 years old revealed that BPA levels negatively correlated with PSA levels in the younger patients but not the non-PCa patients While this observation needs to be validated in a larger clinical study to reach significance, this has crucial repercussions for young patients who take PSA tests for PCa screening. If exposure to high levels of BPA suppresses their serum levels of PSA, this may result in a misdiagnosis. This problem is similar to the under-detection of PCa in hypogonadal men because of the androgen dependency of PSA [45], [46], [47]. The inhibitory effect of BPA may be indirect, acting through the hypothalamic pituitary testicular axis [48]. Alternatively, it might be a direct inhibition on the cancer cells, similar to a report of direct suppression by genistein of PSA production [49].

BPA is not a recognized carcinogen. The question thus arises as to the mechanism behind the positive correlation of BPA exposure with PCa. Several studies have shown that centrosome amplification is a major contributing factor to aneuploidy in human tumors [15], [16]. We hence examined the centrosome profile of PCa cells treated with BPA and found that treatment with BPA increased the number of cells with abnormal centrosomes. One can speculate that BPA may be affecting the cell-cycle machinery involved in centrosome duplication or the structural components required for centrosome duplication and maturation [50], [51]. Perturbations in these events have the potential to induce CA and increase genomic instability. Moreover, the estrogenic action of BPA may affect the expression of genes regulating centrosome cycle. For example, while *AurkA* is not a specific direct target of estrogen *in vitro*, *AurkA* is implicated in estrogen-induced oncogenesis, with long-term treatment of rats with estrogen having been shown to upregulate its expression [52]. Thus, the mechanism by which BPA deregulates the centrosome cycle and induces CA needs further clarification.

An interesting finding was of the greatest sensitivity of the immortalized normal prostate epithelial cell line to the effects of low-dose BPA (Table 3), suggesting that BPA might perturb the centrosome cycle in normal cells and contribute towards aneuploidy. This result is similar to that of previously published studies indicating that a BPA-related increase of DNA adducts was more pronounced in a non-tumorigenic epithelial cell line (PNT1) than in PC3 metastatic carcinoma cells [34]. On the whole, these experimental findings support the hypothesis that BPA plays a role in prostate carcinogenesis, in addition to promoting disease progression.

Another intriguing observation was the non-monotonic response observed in immortalized normal epithelial cells (NPrEC, RWPE-1) and androgen-dependent PCa (LNCaP) cells, suggesting that low concentrations of BPA elicit CA, with the greatest effect at 0.1 nM. This concentration is at least 10- to100-fold lower than most studies reporting a low-dose effect of BPA *in vitro* [53], [54]. At higher BPA concentrations, the detrimental effects on centrosomes appear to disappear. This observation could be explained by findings in the literature that BPA differentially interacts with various receptors such as estrogen receptors α and β, GPR30, or ERRγ, depending on the cell context [55], [56], [57], [58], [59], [60]. Alternatively, it may be a result of checkpoint mechanisms activated, blocking CA at higher BPA doses, causing either cell-cycle arrest or death of cells with dysregulated centrosome duplication. Future studies needs to address the underlying cause of non-monotonic dose-responses in these cell lines.

We found increased MT aster formation in RWPE-1 cells in the presence of BPA. The interphase MT dynamics tightly regulates mitosis. It also maintains normal subcellular localization of organelles, vesicular transport, cell migration, and the overall directionality of cells within the milieu of tissue architecture. In this context, androgen receptor (AR) nuclear localization has been shown to be dependent on the MTs [61], [62]. Since AR nuclear localization is essential for its transcriptional activity [63], it would be interesting to determine whether BPA induced perturbations in MT dynamics impacts AR trafficking and nuclear translocation, and hence alters AR functionality. Moreover, both AR and BPA directly interact with tubulin [62], [64], [65]. One can thus speculate that BPA and AR may compete for tubulin, thus affecting the function of AR. Alternatively, the effects of BPA on MT-dynamics may increase the translocation of AR to the nucleus. Thus, studies on AR trafficking in response to BPA need to be performed, especially in light of reports on the adverse effects of MT-disrupting chemotherapeutic drugs on AR accumulation in nucleus [62]. Hence it is possible that in the non-tumorigenic cells, BPA may initiate or promote PCa progression by interfering with AR function. A previous report has shown that treatment with BPA stimulates human PCa cell migration [33] and affects MT dynamics [66]. Moreover, a change in MT dynamics could be linked to our observation that BPA increased cloning efficiencies of C4-2 cells in soft agar, which could be indicative of enhanced tumorigenicity and/or aggressiveness for these cells *in vivo*. This latter finding supports the notion that BPA may promote PCa progression in addition to its speculative role in neoplastic transformation.

The centrosome is emerging as a potential therapeutic target of drugs in castration resistant PCa (CRPC). Targeted inhibitory compounds are available for inhibition of kinases such as Polo-like kinases, Cyclin-dependent kinases, Aurora kinases, as well as molecular motor proteins [67], some of which have progressed to early clinical trials [68], [69]. Recently, histone deacetylases HDAC1, HDAC5 and SIRT1 have been identified to suppress centrosome duplication and amplification [70], suggesting that HDAC activation could be an important therapeutic avenue in CRPC. Aryl hydrocarbon receptor agonists such as indirubins also reduced centriole overduplication, implying involvement of aryl hydrocarbon receptor signaling in the centrosome cycle [71]. Additionally, the MT-disrupting agents are first line treatments for CRPC [72]. However, because of the ubiquitous presence of BPA, the possible adverse interactions of BPA with these centrosome and MT targeting drugs necessitate evaluation for CRPC.

In short, our findings provide the first evidence that urinary BPA level may have prognostic value for PCa and that disruption of the centrosome duplication cycle by low-dose BPA is a previously unknown mechanism underlying neoplastic transformation and cancer progression in the prostate.
